# Schizotypy and mindfulness: Magical thinking without suspiciousness characterizes mindfulness meditators

**DOI:** 10.1016/j.scog.2016.05.001

**Published:** 2016-05-31

**Authors:** Elena Antonova, Kavitha Amaratunga, Bernice Wright, Ulrich Ettinger, Veena Kumari

**Affiliations:** a King's College London, Institute of Psychiatry, Psychology and Neuroscience (IoPPN), Department of Psychology, London, UK; b University of Bonn, Department of Psychology, Bonn, Germany; c NIHR Biomedical Research Centre for Mental Health, South London and Maudsley NHS Foundation Trust, London, UK

**Keywords:** Schizotypy, Mindfulness, Suspiciousness, Social anxiety

## Abstract

Despite growing evidence for demonstrated efficacy of mindfulness in various disorders, there is a continuous concern about the relationship between mindfulness practice and psychosis. As schizotypy is part of the psychosis spectrum, we examined the relationship between long-term mindfulness practice and schizotypy in two independent studies. Study 1 included 24 experienced mindfulness practitioners (19 males) from the Buddhist tradition (meditators) and 24 meditation-naïve individuals (all males). Study 2 consisted of 28 meditators and 28 meditation-naïve individuals (all males). All participants completed the Schizotypal Personality Questionnaire (Raine, 1991), a self-report scale containing 9 subscales (ideas of reference, excessive social anxiety, magical thinking, unusual perceptual experiences, odd/eccentric behavior, no close friends, odd speech, constricted affect, suspiciousness). Participants of study 2 also completed the Five-Facet Mindfulness Questionnaire which assesses observing (Observe), describing (Describe), acting with awareness (Awareness), non-judging of (Non-judgment) and non-reactivity to inner experience (Non-reactivity) facets of trait mindfulness. In both studies, meditators scored significantly lower on suspiciousness and higher on magical thinking compared to meditation-naïve individuals and showed a trend towards lower scores on excessive social anxiety. Excessive social anxiety correlated negatively with Awareness and Non-judgment; and suspiciousness with Awareness, Non-judgment and Non-reactivity facets across both groups. The two groups did not differ in their total schizotypy score. We conclude that mindfulness practice is not associated with an overall increase in schizotypal traits. Instead, the pattern suggests that mindfulness meditation, particularly with an emphasis on the Awareness, Non-judgment and Non-reactivity aspects, may help to reduce suspiciousness and excessive social anxiety.

## 1 Introduction

*Mindfulness* is a translation of the Pali term *sati* that in meditation context refers to remembering to keep awareness of one's practice. *Mindfulness* practice normally proceeds in stages, starting from the mindfulness of bodily sensations to awareness of feelings and thoughts, ultimately aimed at developing a present-centered awareness without an explicit focus. These stages are apparent in most schools of Buddhism, as well as in Mindfulness-Based Interventions (MBIs) such as Mindfulness-Based Stress Reduction (MBSR; Kabat-Zinn, 1990) and Mindfulness-Based Cognitive Therapy (MBCT; Segal et al., 2002). Mindfulness practice as incorporated in MBIs is often contrasted with more effortful concentration-based practices such as those taught in Theravada Buddhism (Bishop et al., 2004). The traditions of Buddhism most closely aligned with mindfulness as taught in MBSR and MBCT are Dzogchen and Mahamudra, which take a gentle approach to practice by letting go of any striving to achieve a particular mental state, and simply resting in a present-centered awareness free of emotional reactivity and conceptual elaboration (Dunne, 2011, Kabat-Zinn, 2011).

MBSR has been shown to reduce stress, depression, and anxiety, and to improve general wellbeing in a number of physical and psychological conditions (meta-analysis, Hofmann et al., 2010, Zainal et al., 2013), as well as in healthy populations (meta-analysis, Khoury et al., 2015). MBCT has been reported to prevent depression relapse (Teasdale, 2000), and to be at least as effective as anti-depressants (Kuyken et al., 2015). Mindfulness as a trait also inversely correlates with anxiety and depression in healthy individuals (Baer et al., 2004). Despite this transdiagnostic efficacy of MBIs, the relationship between mindfulness and psychosis is currently unclear.

There are persistent concerns that mindfulness might induce psychosis in vulnerable individuals and even in people with no previous history or known vulnerability to psychosis based on a number of single-case studies that appear to suggest that meditation can induce acute psychotic episodes in individuals with a history of schizophrenia (Walsh and Roche, 1979), as well as in people without a history of psychiatric illness (Sethi and Subhash, 2003, Yorston, 2001). However, as discussed in more detail by Shonin et al. (2014), in all these cases the individuals were involved in intensive meditation retreats, and it is unclear to what extent the meditation practices that the described cases were engaged with are in line with the approach employed in MBIs. A number of MBIs for psychosis conducted to date, although mostly preliminary, suggest that mindfulness practice of short duration can actually alleviate the distress associated with psychotic symptoms, such as hearing voices, and reduce depression and anxiety (Abba et al., 2008, Chadwick et al., 2008, Chadwick et al., 2009, Escudero-Perez et al., 2015, Moritz et al., 2015, Randal et al., 2015, Strauss et al., 2015, Tong et al., 2015, Ubeda-Gomez et al., 2015).

With a clinical prevalence of about 7 per 1000 in the adult population, psychosis is more common among the general population than previously assumed (Johns et al., 2004) and is expressed along a continuum (Verdoux and Van Os, 2002). Schizotypy is a psychological construct, encompassing a range of personality traits and cognitions that are similar to psychosis but less severe in nature (Ettinger et al., 2014). According to Raine et al. (1995), schizotypy is characterized by nine dimensions: ideas of reference, excessive social anxiety, magical thinking, unusual perceptual experiences, eccentric behavior or appearance, no close friends or confidants, odd speech, constricted affect and suspiciousness. Schizotypy clearly encompasses both psychosis-like symptoms and symptoms related to anxiety and depression.

The main aim of the present study therefore was to examine the relationship between regular long-term (> 2 years) practice of mindfulness and the dimensions of schizotypy (Raine, 1991) in two independent studies. Based on the reviewed evidence for the positive effects of mindfulness on anxiety and depression, it was hypothesized that experienced meditators will score lower on the excessive social anxiety and constricted affect compared to meditation-naïve individuals. Given the lack of any direct data on this topic, no specific predictions were made in relation to other schizotypy dimensions. It was, however, anticipated that any associations present in both studies, even if with a small effect size, would represent true effects. Study 2, in addition to aiming to replicate the findings of Study 1, explored the relationship between the dimensions of schizotypy and the facets of trait mindfulness indexed by the Five Facets Mindfulness questionnaire (FFMQ) (Baer et al., 2006).

## 2 Methods

### 2.1 Participants and design

This investigation included two independent studies. Study 1 included 24 experienced lay meditators (19 males) and 24 meditation-naïve individuals (all males). The meditators were recruited from Buddhist centers across the UK via posters and advertisements. Meditators had to have been consistent in their practice for over 2 years, practicing at least 6 days a week for a minimum of 45 min a day, and were drawn from Dzogchen and Mahamudra traditions of Tibetan Buddhism. Meditation-naïve individuals had to have no experience of mindfulness-related practices including meditation, yoga, tai chi, chi gong, or martial arts and were recruited from a database of healthy volunteers as well as emails and circulars sent to the students and staff of King's College London. Study 2 included 28 experienced male meditators mainly from Zen, Theravada, Vajrayana and Triratna traditions of Buddhism, and 28 meditation-naïve male individuals, recruited in the same way as Study 1 using the same criteria.

Additional inclusion criteria for both studies included IQ > 80 as assessed by Wechsler Abbreviated Scale of Intelligence (Wechsler, 1999), age between 18 and 60 years, non-smoking and not drinking more than 28 units of alcohol per week. Participants with diagnosis of neuropsychiatric disorders, current or past, substance abuse and/or regular prescription medication as assessed by the screening interview were excluded.

The study procedures were approved by King's College London research ethics committee. Participants provided written informed consent to their participation and were compensated for their time.

### 2.2 Assessment of schizotypal personality traits and mindfulness

All participants completed the Schizotypal Personality Questionnaire (SPQ) (Raine, 1991) which contains 9 subscales: ideas of reference, excessive social anxiety, magical thinking, unusual perceptual experiences, odd/eccentric behavior, no close friends, odd speech, constricted affect and suspiciousness. This 74-item assessment of DSM-III-R schizotypal personality disorder provides an overall score of individual differences in schizotypal personality in addition to the scores of the above-mentioned subscales. With high internal reliability (0.90), test–retest reliability (0.82), convergent validity (0.59) and discriminant and criterion validity (0.63, 0.68), it is considered a well-validated measure of schizotypy.

All participants of Study 2 also completed the FFMQ (Baer et al., 2006) to investigate the relationship between trait mindfullness and schizotypy. FFMQ has been derived from factor analysis performed on five of the most commonly used mindfulness measures. The five facets are observing (Observe), describing (Describe), acting with awareness (Awareness), non-judging of inner experience (Non-judgment), and non-reactivity to inner experience (Non-reactivity) as assessed using Likert scale with 39 items. FFMQ has high internal consistency, ranging from 0.75 (Non-reactivity) to 0.91 (Describe).

### 2.3 Data analysis

Group differences in age, IQ, FFMQ and SPQ scores were examined using independent sample t-tests, run separately for the two studies. Given the significant difference in age and IQ between the meditator and meditation-naïve groups in Study 1 (Table 1), we examined the relationship of SPQ scores with age and IQ, and then re-evaluated the group difference in one of the SPQ subscales (no close friends) that showed a positive association with IQ (in Study 1), using analysis of covariance (ANCOVA) co-varying for IQ.

**Table 1:** Demographic characteristics, trait mindfulness and schizotypal scores of the meditator and meditation-naïve groups.

	Study 1			Study 2		
	Meditators Mean (SD)	Meditation-naive Mean (SD)	Group difference t (df = 48), (p)	Meditators Mean (SD)	Meditation-naive Mean (SD)	Group difference t (df = 58), (p)
Age (years)	46.5 (10.49)	39.08 (9.66)	2.54 (**0.01**)	39.68 (10.19)	36.68 (8.41)	1.20 (0.23)
IQ	126.5 (3.89)	120.75 (10.81)	2.40 (**0.04**)	117.46 (13.95)	117.92 (11.57)	0.13 (0.89)
Meditation practice (years)	14.59 (14.41)			9.63 (7.15)		-
**Mindfulness (FFMQ) scores**						
Observe				30.64 (4.53)	25.78 (5.64)	3.55 (**0.001**)
Describe				30.43 (3.66)	30.28 (4.85)	1.12 (0.19)
Awareness				29.90 (5.37)	28.00 (4.52)	1.43 (0.16)
Non-judgment				33.46 (4.56)	27.82 (5.46)	4.20 **(< 0.001**)
Non-reactivity				26.36 (4.82)	21.93 (3.02)	4.12 **(< 0.001**)
**Schizotypy (SPQ) scores**						
Ideas of reference	1.40 (1.41)	1.50 (1.50)	1.19 (0.84)	1.82 (1.89)	1.75 (2.08)	0.13 (0.89)
Excessive social anxiety	1.29 (1.27)	1.92 (1.44)	1.52 (***0.12***)	1.32 (1.36)	2.18 (1.89)	1.94 (***0.056***)
Magical thinking	3.08 (1.79)	1.12 (1.67)	3.91 **(< 0.001**)	2.25 (2.44)	0.96 (1.64)	2.31 (**0.025**)
Unusual perceptual experiences	1.62 (1.44)	1.33 (1.71)	0.64 (0.53)	1.61 (1.75)	1.29 (1.54)	0.73 (0.47)
Odd/eccentric behavior	1.50 (1.38)	1.87 (1.75)	0.73 (0.47)	1.43 (1.62)	0.79 (1.34)	1.62 (0.11)
No close friends	1.17 (1.40)	2.33 (2.40)	2.05 (0.05)⁎	1.25 (1.38)	0.96 (1.37)	0.78 (0.44)
Odd speech	1.42 (1.47)	2.37 (2.32)	1.71 (0.09)	2.11 (1.52)	2.36 (1.06)	0.52 (0.61)
Constricted affect	1.08 (1.10)	2.12 (2.03)	2.21 (**0.03**)	1.00 (1.25)	1.07 (1.12)	0.22 (0.82)
Suspiciousness	0.62 (0.77)	1.25 (1.33)	1.99 (**0.05**)	0.71 (0.94)	1.43 (1.64)	2.00 (**0.05**)
Total SPQ score	11.62 (6.38)	14.50 (8.82)	1.29 (0.20)	11.50 (8.68)	11.89 (7.82)	0.18 (0.86)

⁎ Not significant after controlling for IQ.

In Study 2, we examined the correlations between trait mindfulness (FFMQ) and SPQ (total and subscale) scores across both samples, and then separately in the meditator and meditation-naïve groups. Given the limited range of scores on some SPQ subscales, we report Spearman correlations (the same pattern of associations was observed with Pearson's *r*).

All data analysis was conducted using IBM Statistical Package for Social Sciences (version 22). The alpha level of significance (two-tailed) was set at p = 0.05 in all analyses unless specified otherwise.

## 3 Results

Demographic characteristics of the meditator and meditation-naïve groups, along with the descriptive statistics and group differences in SPQ and FFMQ scores, are presented in Table 1.

### 3.1 Study 1

Meditators were older and had higher IQ than meditation-naïve individuals (Table 1). Meditators scored significantly higher on ‘magical thinking’ and significantly lower on the ‘suspiciousness’, ‘constricted affect’ and ‘no close friends’ subscales of the SPQ relative to meditation-naïve individuals. The two groups did not differ in total schizotypy scores (Table 1). Unexpectedly, there was a significant negative correlation between IQ and ‘no close friends’ subscale scores (across all participants, r = − 0.324, p = 0.04) and the significant difference between the meditator and meditation-naïve groups in ‘no close friends’ scores was abolished when we controlled for IQ (p > 0.20). IQ and age were not correlated with ‘excessive social anxiety’, ‘magical thinking’ or ‘suspiciousness’ (or with any other SPQ subscale) scores.

### 3.2 Study 2

The meditator and meditation-naïve groups were comparable on age and IQ (Table 1). Replicating the observations of Study 1, meditators scored significantly higher on ‘magical thinking’ and lower on ‘suspiciousness’ relative to meditation-naïve individuals. They also scored lower, at trend-level, on ‘excessive social anxiety’ (a weak trend also present in Study 1). As in Study 1, total SPQ profile did not significantly differ between the two groups (Table 1). Age and IQ did not correlate with SPQ (total or subscale) scores.

Meditators scored significantly higher on the Observe, Non-judgment and Non-reactivity mindfulness facets of FFMQ compared to meditation-naïve individuals (Table 1). Across all participants (n = 56), there were negative correlations of ‘excessive social anxiety’ with Awareness and Non-judgment; ‘odd speech’ with Describe and Awareness; ‘constricted affect’ with Awareness and Non-judgment; and ‘suspiciousness’ with Awareness, Non-judgment and Non-reactivity (Table 2). Both the meditator and meditation-naïve groups contributed to all these relationships, except for the negative correlation between suspiciousness and Non-reactivity, which was present mainly in the meditator group (Table 2).

**Table 2:** Spearman rank order correlations between mindfulness facets (FFMQ) and schizotypy (SPQ) dimensions in Study 2.

Both samples	SPQ									
FFMQ	Ideas of reference	Excessive social anxiety	Magical thinking	Unusual perceptual experiences	Odd/eccentric behavior	No close friends	Odd speech	Constricted affect	Suspiciousness	Total SPQ scores
Observe	− 0.013	− 0.135	0.219	− 0.010	0.108	− 0.200	− 0.080	− 0.147	− 0.087	− 0.025
Describe	− 0.083	− 0.118	0.122	0.029	0.130	0.032	− 0.280*	− 0.178	− 0.117	− 0.058
Awareness	− 0.155	− 0.428***	0.045	− 0.067	0.075	− 0.134	− 0.548***	− 0.260*	− 0.298*	− 0.320**
Non-judgment	− 0.227	− 0.374**	− 0.014	− 0.174	− 0.070	− 0.109	− 0.164	− 0.282*	− 0.469***	− 0.348**
Non-reactivity	− 0.074	− 0.152	0.180	− 0.021	0.020	− 0.037	− 0.136	0.047	− 0.304*	− 0.052
**Meditators only**										
Observe	− 0.177	− 0.368*	0.123	− 0.287	− 0.121	− 0.478**	− 0.523**	− 0.349	− 0.150	− 0.377*
Describe	0.042	0.011	0.314	0.150	0.213	0.050	− 0.253	0.034	0.066	0.185
Awareness	− 0.158	− 0.384*	0.018	− 0.148	− 0.057	− 0.259	− 0.591***	− 0.175	− 0.204	− 0.295
Non-judgment	− 0.139	− 0.369*	0.043	− 0.221	− 0.338	− 0.073	− 0.129	− 0.123	− 0.389*	− 0.261
Non-reactivity	− 0.192	− 0.355	0.045	− 0.233	− 0.206	− 0.250	− 0.434*	− 0.125	− 0.338	− 0.275

Meditation-naïve only										
Observe	0.089	0.219	0.383*	0.127	0.191	0.139	0.299	0.071	0.180	0.293
Describe	− 0.118	− 0.238	0.001	− 0.065	0.103	− 0.085	**− 0.304**	− 0.389*	− 0.315	− 0.258
Awareness	− 0.141	− 0.420*	0.039	0.010	0.145	− 0.011	− 0.575***	− 0.345	− 0.309	− 0.371*
Non-judgment	− 0.408*	**− 0.309**	− 0.271	− 0.338	− 0.038	− 0.346	− 0.258	− 0.439*	− 0.354	− 0.552**
Non-reactivity	− 0.102	0.309	0.033	0.129	0.083	0.081	0.139	0.312	− 0.072	0.126

*p ≤ 0.05; ** p ≤ 0.01; ***p ≤ 0.001.

## 4 Discussion

In line with our a priori hypothesis, meditators, compared to the meditation-naïve individuals, scored significantly lower on ‘constricted affect’ in Study 1, and showed a trend level for lower scores on ‘excessive social anxiety’ in both studies. In addition, meditators scored significantly higher on ‘magical thinking’, and significantly lower on ‘suspiciousness’ in both studies. In relation to the association between (FFMQ) trait mindfulness and (SPQ) schizotypy dimensions (Study 2), lower ‘excessive social anxiety’ and ‘constricted affect’ scores were associated with higher Awareness and Non-judgment; lower ‘odd speech’ with higher Awareness and Describe; and lower ‘suspiciousness’ with higher Awareness, Non-judgment and Non-reactivity scores.

‘Constricted affect’ relating to a form of emotional blunting (Raine, 1991) appears to be positively affected only by the mindfulness practice styles of Dzogchen and Mahamudra (Study 1) which are most similar to the MBSR/MBCT approach, as this schizotypy dimension did not significantly differentiate the long-term meditators drawn from Zen, Vipassana, Theravada, Vajrayana and Triratna traditions of Buddhism from the meditation-naïve participants in Study 2. The ‘excessive social anxiety’ subscale relates to overt physiological changes along with a high degree of nervousness and anxiety (Raine, 1991). The finding of lower scores in meditators on this subscale, albeit non-significant, is in line with the notion that mindfulness training reduces anxiety (e.g., Khoury et al., 2015). Significant inverse correlations of lower ‘excessive social anxiety’ and ‘constricted affect’ scores with higher Awareness and Non-judgment scores suggest that mindfulness trait alleviates the so called negative symptoms of schizotypy via non-judgemental present-centered awareness, and this effect could be strengthened by mindfulness practice as suggested by significantly higher scores on the Non-judgment facet in long-term meditators, compared to mediation-naïve individuals. This is in line with preliminary evidence showing ameliorating effects of mindfulness training on symptoms of anxiety and depression in people with psychosis by reducing self-critical attitudes and developing non-judgmental present-centered awareness, as well as self-acceptance and self-compassion (review, Shonin et al., 2014).

One of our novel findings is that meditators scored significantly lower on ‘suspiciousness’ in both samples. Although not specifically hypothesized, this finding is highly relevant to the clinical applications of mindfulness for the prevention and treatment of psychosis. From the time of Kraepelin, suspiciousness and paranoia have been considered to be among the main symptoms of psychosis (Kendler et al., 1996). These symptoms may stem from the avoidance of personal exposure and negative self-image, distorting reality in the process so as to strengthen impaired self-esteem (Bentall et al., 2008, Oxman et al., 1982). This avoidant nature is in contrast to mindfulness, which promotes direct engagement with reality and attention to all aspects of the present-moment experience non-judgmentally and non-reactively. Similarly, the distorted view of one-self and the characteristics of suspiciousness and paranoia are in contrast to greater empathy, compassion, and prosocial behavior associated with mindfulness (Condon et al., 2013). Given that a) paranoid schizophrenia is the most common type of psychosis experienced (Lieberman et al., 2012), b) suspiciousness/paranoia carries a high predictive power for conversion to psychosis in high risk individuals, alongside genetic risk and unusual thought content (Cannon et al., 2008), and c) we found inverse correlation between ‘suspiciousness’ and Non-judgement in meditators only, MBIs might hold promise in preventing psychosis in high-risk individuals.

Another novel finding of our investigation is higher score on ‘magical thinking’ in meditators in both studies. Given that ‘magical thinking’ was not associated with any of the FFMQ facets that were significantly higher in meditators compared to meditation-naïve individuals in study 2 and, as such, does not appear to develop due to mindfulness practice per se, the most likely explanation for this finding is that our mindfulness meditators were mainly practicing within Buddhist tradition. The ‘magical thinking’ subscale measures beliefs into such supernatural experiences as telepathy, clairvoyance, astrology, and sixth sense, which are incorporated into Buddhist psychology and metaphysics, particularly in the Tibetan Buddhist tradition. The higher scores on ‘magical thinking’ in the face of low scores on other schizotypy dimensions are in line with research showing that having a context for unusual experiences and/or beliefs makes a difference in terms of whether they lead to diagnosable mental health difficulties or whether they become integrated into one's life without causing a functional disruption (Brett et al., 2014, Heriot-Maitland et al., 2012, Peters et al., 2016). It is also possible that people attracted to meditation practice within the context of Buddhist beliefs and metaphysics are higher on magical thinking to begin with, or that higher score on ‘magical thinking’ simply reflects greater openness to experience in meditators, rather than actual beliefs in these ‘supernatural’ constructs. The latter possibility is more likely given that trait mindfulness has been shown to be associated with greater openness to experience (Baer et al., 2004, van den Hurk et al., 2011), and there is an association between schizotypy and openness to experience (DeYoung et al., 2011). Mindfulness meditators thus may simply have greater open-mindedness towards what constitutes ‘magical thinking’ in the SPQ than the average population. Whether higher ‘magical thinking’ is an ‘artifact’ of Buddhist belief system or whether it indexes greater open-mindedness of mindfulness practitioners could be addressed by further research by recruiting long-term meditators that practice mindfulness within a secular setting.

Particularly relevant to psychosis is our finding that higher ‘magical thinking’ in meditators was not accompanied with higher ‘ideas of reference’. The ‘ideas of reference’ subscale measures the tendency to self-reference the experience, i.e. over-subscribe personal relevance and meaning to inner experiences and external events. Mindfulness practice, on the other hand, attenuates self-referential tendencies and associated brain dynamics (Brewer et al., 2011, Farb et al., 2007); the same brain networks are found to be hyperactive in people with schizophrenia (Whitfield-Gabrieli et al., 2009). The combination of high magical thinking and low ideas of reference is in alignment with the frameworks of psychosis that suggest that it is not unusual beliefs and/or experiences per se that constitute a risk for psychosis, it is rather their interpretation and hyper self-referencing (Peters et al., 2012). Given that unusual beliefs and thought content constitute risk for psychosis conversion (Cannon et al., 2008), the reduction of self-referencing might be another rationale for mindfulness-based psychosis prevention.

The observed pattern of inverse associations between the dimensions of schizotypy (‘excessive social anxiety’, ‘odd speech’, ‘constricted affect’ and ‘suspiciousness’) and the Awareness, Non-judgment and Non-reactivity facets of mindfulness suggests that trait mindfulness reduces negative dimensions of schizotypy, whereas mindfulness practice might have further ameliorating effects on ‘excessive social anxiety’ and ‘suspiciousness’ as these were lower in meditators compared to meditation-naïve participants. These findings may have important therapeutic implications, suggesting that a) future MBIs with a strong emphasis on the Awareness, Non-judgment and Non-reactivity aspects of mindfulness may be particularly effective in reducing anxiety-related symptoms, depression, and suspiciousness in psychosis; and b) mindfulness could be used as a therapeutic tool for psychosis prevention by addressing suspiciousness and paranoia in high risk populations.

Our investigation has a number of limitations. First, it examined the relationship between schizotypy dimensions and mindfulness in a cross-sectional correlational design, without any knowledge of the meditators' schizotypy scores prior to them starting mindfulness practice. Future research could examine the effects of shorter duration MBIs on the relationship between these traits. Second, this investigation was opportunistic, using two existing data sets (from two independent psychophysiology projects, Antonova et al., 2015, Kumari et al., 2015), consisting of mostly (Study 1) or only men (Study 2). Our findings thus cannot be generalized to women. Third, both schizotypy and mindfulness were assessed using self-report methods. While self-reports have their strengths, such as in-depth, detailed data gathered directly from the participant, whilst limiting experimenters' bias, they also have limitations, such as socially desired responses resulting in underestimation or overestimation of actual traits. Given that people's perceptions of themselves are known to be poor predictors of their behavior (Baumeister et al., 2007), future studies, wherever possible, should incorporate experimental analogues of relevant phenomena (e.g. Atherton et al., 2016).

In conclusion, to our knowledge, this is the first investigation to have focused on the schizotypy profiles of experienced mindfulness practitioners. The findings demonstrated lower (trend-level) 'excessive social anxiety', as well as significantly lower 'suspiciousness' and higher 'magical thinking' in meditators relative to meditation-naïve individuals. These differences, taken together with the pattern of correlational observations (i.e. inverse associations between 'excessive social anxiety' and Awareness and Non-judgment; and between 'suspiciousness' and Awareness, Non-judgment and Non-reactivity), suggest that mindfulness training with emphasis on developing the facets of Awareness, Non-judgment and Non-reactivity may help to reduce social anxiety and suspiciousness in psychosis and related populations.

## Role of funding source

The sponsors had no role in study design; in the collection, analysis and interpretation of data; in the writing of the report; or in the decision to submit the paper for publication.

## Contributors

Elena Antonova and Veena Kumari conceptualized the study. Elena Antonova and Bernice Wright assisted with participant recruitment and data collection. Veena Kumari and Elena Antonova undertook the statistical analysis and prepared the first draft. All authors contributed to the final version.

## Conflict of interest

The authors report no biomedical financial interests or potential conflicts of interest.
